# Diagnosis-specific readmission risk prediction using electronic health data: a retrospective cohort study

**DOI:** 10.1186/1472-6947-14-65

**Published:** 2014-08-04

**Authors:** Courtney Hebert, Chaitanya Shivade, Randi Foraker, Jared Wasserman, Caryn Roth, Hagop Mekhjian, Stanley Lemeshow, Peter Embi

**Affiliations:** 1 Department of Biomedical Informatics, The Ohio State University, Columbus, OH, USA; 2 Division of Infectious Diseases, The Ohio State University, Columbus, OH, USA; 3 Department of Computer Science and Engineering, The Ohio State University, Columbus, OH, USA; 4 College of Public Health, The Ohio State University, Columbus, OH, USA; 5 Division of Gastroenterology, Hepatology & Nutrition, The Ohio State University, Columbus, OH, USA; 6 Division of Immunology and Rheumatology, The Ohio State University, Columbus, OH, USA; 7 The Dartmouth Institute of Health Policy and Clinical Practice, Lebanon, NH, USA

**Keywords:** Readmissions, Risk-prediction, Electronic health records

## Abstract

**Background:**

Readmissions after hospital discharge are a common occurrence and are costly for both hospitals and patients. Previous attempts to create universal risk prediction models for readmission have not met with success. In this study we leveraged a comprehensive electronic health record to create readmission-risk models that were institution- and patient- specific in an attempt to improve our ability to predict readmission.

**Methods:**

This is a retrospective cohort study performed at a large midwestern tertiary care medical center. All patients with a primary discharge diagnosis of congestive heart failure, acute myocardial infarction or pneumonia over a two-year time period were included in the analysis.

The main outcome was 30-day readmission. Demographic, comorbidity, laboratory, and medication data were collected on all patients from a comprehensive information warehouse. Using multivariable analysis with stepwise removal we created three risk disease-specific risk prediction models and a combined model. These models were then validated on separate cohorts.

**Results:**

3572 patients were included in the derivation cohort. Overall there was a 16.2% readmission rate. The acute myocardial infarction and pneumonia readmission-risk models performed well on a random sample validation cohort (AUC range 0.73 to 0.76) but less well on a historical validation cohort (AUC 0.66 for both). The congestive heart failure model performed poorly on both validation cohorts (AUC 0.63 and 0.64).

**Conclusions:**

The readmission-risk models for acute myocardial infarction and pneumonia validated well on a contemporary cohort, but not as well on a historical cohort, suggesting that models such as these need to be continuously trained and adjusted to respond to local trends. The poor performance of the congestive heart failure model may suggest that for chronic disease conditions social and behavioral variables are of greater importance and improved documentation of these variables within the electronic health record should be encouraged.

## Background

Readmissions are a widespread and costly problem for hospitals across the United States [1-4]. In 2012, the average rate of 30-day readmission for Medicare patients was 24.7% for congestive heart failure (CHF), 18.5% for pneumonia (PNA) and 19.8% for acute myocardial infarction (AMI) [5]. There are many incentives for reducing readmission rates from a financial and quality-of-care perspective [6-9]. However, interventions can be time- and cost-intensive [10-14] and it may not be cost-effective to intervene upon every patient regardless of his or her risk of readmission. Traditionally, healthcare providers do a poor job of predicting which patients will be readmitted [15].

Several studies have used administrative and clinical data to identify predictors of readmission for CHF, PNA and AMI [16-20], however, few patient-level characteristics are consistently associated with risk of readmission [21-26] and most prediction models perform poorly [27]. Amarasingham and colleagues developed a prediction model based on local data, which performed better than models developed for general use [23]. Even though high readmission rates are seen in hospitals across the country [4], data suggest that differences may exist between 30-day readmission rates in different settings [2,4,8], indicating that geographic and socioeconomic factors may affect the likelihood of readmission.

With this in mind, we created prediction models at our own institution, The Ohio State University Wexner Medical Center (OSUWMC) that are specific to our patients, their context, and specific disease state. We developed prediction models for 30-day readmissions that examined previously studied, as well as novel variables. We used variables available in our Information Warehouse (IW) to build readmission prediction models for CHF, PNA, AMI and a combined model that included all three groups. We hypothesized that a model tuned to a specific disease state would perform better than a combined model, and that a model created at our own institution would be uniquely suited for our patient population and environment. These models are the first step in a plan to embed a tool into our comprehensive electronic health record (EHR) to alert physicians to high-risk patients at the point-of-care.

## Methods

### Settings and participants

This was a retrospective study using two years of data collected from the IW at the OSUWMC. The IW captures all administrative and clinical data during inpatient hospitalizations. Eligible patients were those admitted to an inpatient service between August 1, 2009 and July 31, 2011 with a primary discharge diagnosis International Classification of Diseases, Version 9 Clinical modification (ICD-9-CM) code of CHF, PNA, or AMI, as defined by the Centers for Medicare and Medicaid Services (CMS) [28].

### Definitions

**Figure 1:**
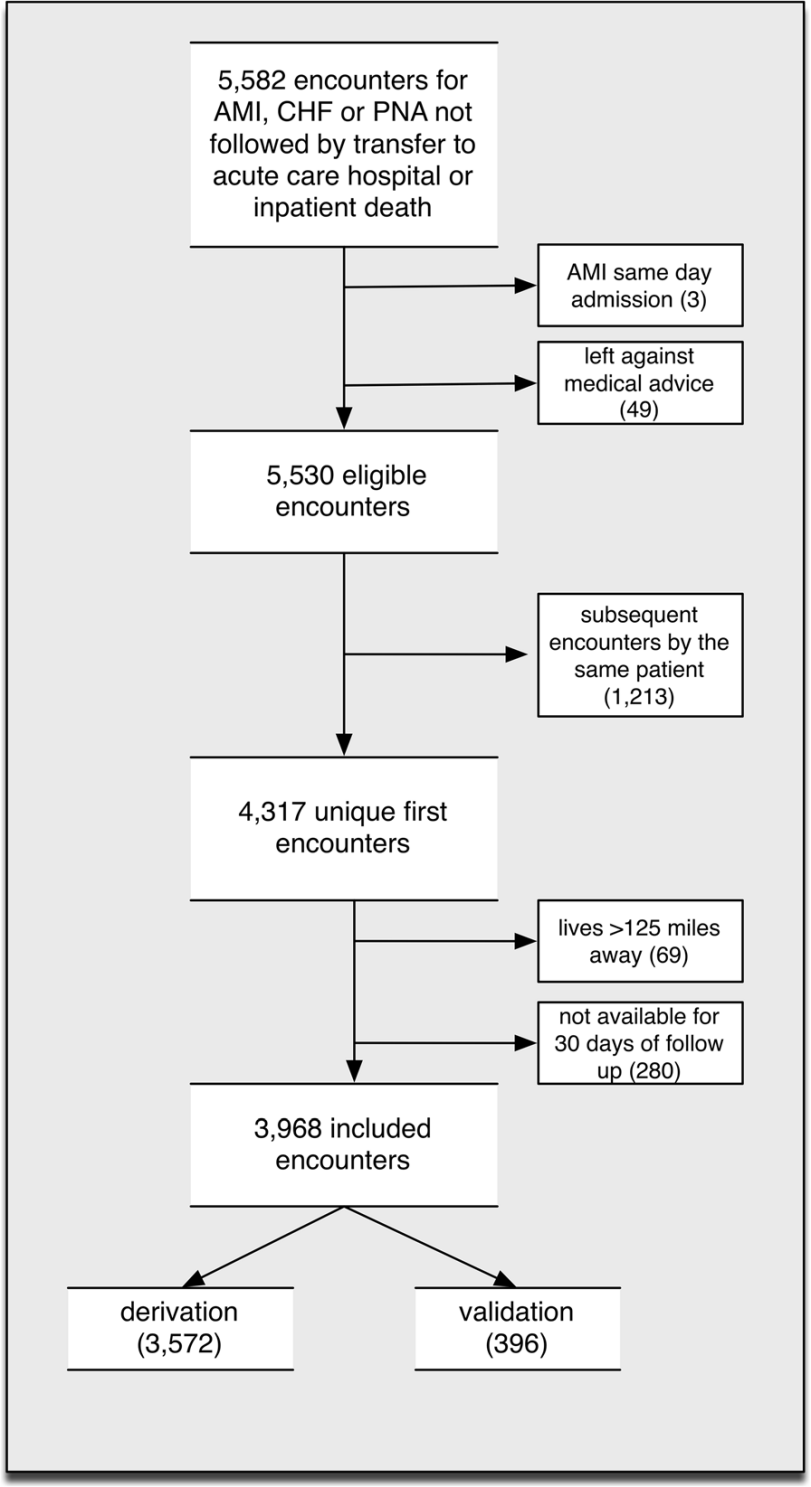
Inclusion and exclusion criteria for derivation and random sample validation cohorts.

We defined an index hospitalization as the first hospitalization for CHF, PNA, or AMI during the study period. Our query excluded index hospitalizations that were followed by transfer to an acute care setting or resulted in the patient’s death during the hospitalization. We excluded index hospitalizations of patients who left against medical advice (n = 49), as well as admissions that resulted in same-day discharges for AMI (n = 3). We excluded patients without 30-days of follow-up after discharge, including patients who were discharged within 30 days of the end of the study and were not readmitted prior to the end of the study (n = 130) and patients that died within 30 days of discharge without a readmission (n = 150). Only the first admission for each patient was included. An overview of specific inclusion and exclusion criteria can be seen in Figure 1.

We classified a 30-day readmission as an admission to an inpatient service between the index hospitalization discharge date and 30 days after discharge. We considered admission for any cause to be a readmission, with the exception of a planned admission for a coronary artery bypass graft or percutaneous transluminal coronary angioplasty after an index admission for AMI (n = 4). We counted only the first readmission for each patient.

We used zip codes as a proxy for patients’ address of residence. Most patients (74%) lived within 50 miles of OSUWMC, as indicated by the straight-line distance between zip code centroids. 69 patients resided more than 125 miles from the OSUWMC. Because we did not have data on whether patients were readmitted to other hospitals, we used extreme distance as an indicator that these patients would likely be readmitted to outside hospitals if they were readmitted, and as such were substantially different from the rest of the cohort. These 69 patients were excluded as outliers.

### Data collection

We collected administrative and clinical data on all eligible patients. These data included demographics, comorbidities, laboratory results, medication orders, and social history. We identified comorbidities from administrative ICD-9-CM codes associated with the index encounter. We included 31 comorbidities, which we adapted from ICD-9-CM groupings previously published [29]. We also calculated a modified Charlson comorbidity score only using ICD-9-CM associated with the index encounter [29,30]. All data collected were encounter-level data and did not include historical or outpatient data.

The number of medications prescribed on discharge was included as a continuous variable. Medication variables were collected from the list of discharge medications. Laboratory values were classified by the highest and lowest value during the index hospitalization using accepted clinical cut-points. All included laboratory data had less than 1% missing data. We did not include most social history variables (e.g. smoking, employment and living situation), as they were not consistently reported in the EHR. All continuous variables were assessed for appropriate transformations using fractional polynomials.

Because of the small number of patients of races other than white or black, this variable was defined as black versus non-black. Age was treated as a continuous variable. We created a binary variable for marital status, where “single” included those patients classified as divorced, single, widowed, or separated. An inpatient visit in the last 30 days included any admission to an inpatient facility including inpatient rehabilitation, but excluding emergency department (ED) visits that did not result in admission. A variable representing an ED visit in the last 30 days represented those ED visits that were not associated with an inpatient admission.

### Validation

We validated our models in two ways. The first method used a random sample from the original cohort as validation. We maintained the percentage readmitted in these validation samples. Ten percent (n = 396) of patients were randomly removed from the combined model and 20% from the individual models, prior to model creation. The second method used a historical validation cohort. We performed a second, identical data pull for patients admitted between August 1, 2008 and July 31, 2009. We applied the same exclusion criteria, with one addition: if patients were already part of the derivation cohort, they were excluded from the validation cohort (n = 327).

### Statistical analysis

We performed all analyses using Stata (StataCorp. 2011. *Stata Statistical Software: Release 12*. College Station, TX: StataCorp LP). We performed univariate analyses on each variable in the combined derivation cohort and within CHF, PNA, and AMI subsets and included those variables in the regression with a p-value <0.2.

We included eligible variables in a stepwise logistic regression against the binary variable, readmission within 30 days. Variables with a p-value <0.1 were allowed to remain in the model. We estimated odds ratios and 95% confidence intervals for readmission within 30 days. We evaluated the resulting multivariable models using the area under the receiver operating characteristic curves (AUC) and evaluated the goodness-of-fit using the Hosmer-Lemeshow test. We tested for outliers using standardized Pearson residuals, Pregibon’s dbeta and the leverage of each observation [31]. Removing the outliers resulted in minimal changes in the p-value for goodness-of-fit and AUC, thus, they remained in the model.

We applied each of the four derived models to its corresponding validation cohort, using the coefficients from the derivation models. We calculated AUC and evaluated goodness-of-fit. To assess the predictive ability of each of the models, we calculated the logistic function (P(y|x) = 1/(1 + (e^-b0+b1x)^)) for each patient in the derivation cohort, which resulted in a predicted probability of readmission. We divided these probabilities into tertiles of risk (low, medium and high). We then calculated the logistic function for the validation cohort using the same coefficients. We used the same cutoffs for low-, medium-, and high-risk determined by the derivation cohort. We then compared these risks to the actual rate of readmission in each of the groups for the derivation and validation cohorts (Figure 1).

The OSUWMC institutional review board (IRB) approved all data collection. Given that this was a retrospective study of data already collected for clinical purposes, a waiver of informed consent was granted by the IRB.

## Results

### Overall findings

The derivation cohort included 3572 patients; 1354 in CHF, 1171 in PNA and 1047 in AMI. The readmission rates were 16.2% (n = 577) in the combined cohort, 16.4% (n = 222) in CHF, 18.4% (n = 216) in PNA, and 13.3% (n = 139) in AMI. The clinical characteristics of patients included in the analysis are described in Table 1. The mean age was 61 years (IQR 51–72), the majority of patients were male, and there was a high prevalence of comorbidities (data not shown) including diabetes mellitus (DM) (39%), chronic pulmonary disease (42%) and renal disease (28%).

**Table 1:** Characteristics of the study population

	**Derivation cohort N = 3572**	**Historical validation cohort N = 1756**	**Random sample combined cohort N = 396**
**Readmitted in 30 days**	577 (16.2%)	311 (17.7%)	64 (16.2%)
**Demographics**			
Age, median (IQR)	61 (51–72)	62 (52–73)	61 (52–71)
Gender, Female (%)	1541 (43.1%)	759 (43.2%)	162 (40.9%)
Marital status, single* (%)	2023 (56.6%)	988 (56.3%)	203 (51.3%)
Race, Black† (%)	1090 (30.5%)	497 (28.3%)	130 (32.8%)
Distance from the zip code centroid to hospital, miles [median, (IQR)]	9.6 (4.6–46.2)	12 (4.6–53.6)	9.4 (4.6–46.2)
**Comorbidities**			
Charlson Score‡, median (IQR)	3 (1–4)	3 (2–5)	3 (2–4)
Solid tumor	192 (5.4%)	104 (5.9%)	27 (6.8%)
Other neurologic disease	185 (5.2%)	95 (5.4%)	18 (4.6%)
Hypertension	2,533 (70.9%)	1,239 (70.6%)	289 (73.0%)
Lymphoma	86 (2.4%)	46 (2.6%)	8 (2.0%)
Abnormal weight loss	231 (6.5%)	94 (5.4%)	34 (8.6%)
Obesity	550 (15.4%)	258 (14.7%)	67 (16.9%)
Liver disease	154 (4.3%)	61 (3.5%)	18 (4.6%)
Peripheral vascular disease	286 (8.0%)	184 (10.5%)	45 (11.4%)
Arrhythmia	1,149 (32.2%)	722 (41.1%)	145 (36.6%)
Metastatic cancer	106 (3.0%)	74 (4.2%)	15 (3.8%)
**Details of hospital visit**			
Length of stay, median (IQR)	4 (2–7)	4 (3–8)	4 (2–7)
Inpatient visit within the last 30 days	379 (10.6%)	181 (10.3%)	45 (11.4%)
ED visit within the prior 30 days	167 (4.7%)	94 (5.4%)	18 (4.6%)
Number of discharge medications	12 (8–16)	12 (8–16)	12 (8–16)
**AMI**	1047 (29.3%)	594 (33.8%)	119 (30.1%)
**CHF**	1354 (37.9%)	610 (34.7%)	148 (37.4%)
**PNA**	1171 (32.8%)	552 (31.4%)	129 (32.6%)

*Abbreviations*: *IQR* interquartile range, *ED* emergency department, *AMI* acute myocardial infarction, *CHF* congestive heart failure, *PNA* pneumonia.

*Single includes single, widowed, divorced and separated.

†Versus not black.

‡Calculated only using ICD-9 data from the index encounter.

The random-sample validation included 396 (CHF = 148, PNA = 129, AMI = 119) patients in the combined cohort with a readmission rate of 16.2% (n = 64). The CHF cohort had 300 patients with a readmission rate of 16.0% (n = 48); the PNA cohort had 258 patients with a rate of 18.6% (n = 48); and AMI had 230 patients with a rate of 13% (n = 30).

The historical validation cohort consisted of 1756 patients (CHF = 610, PNA =552, AMI = 594) with a combined 30-day readmission rate of 17.7% (n = 311). 19.8% of CHF patients (n = 121), 17.8% of PNA patients (n = 98), and 15.5% of AMI patients (n = 92) were readmitted. The validation cohort was similar to the derivation cohort in all measured patient characteristics (Table 1).

### Univariate analysis

Out of over 100 initial variables, there were 43 that met initial derivation model inclusion criteria (p <0.20) in the combined model. These variables included demographics, comorbidities, laboratory values and certain discharge medications.

### Multivariable analysis

#### Derivation cohort

**Figure 2:**
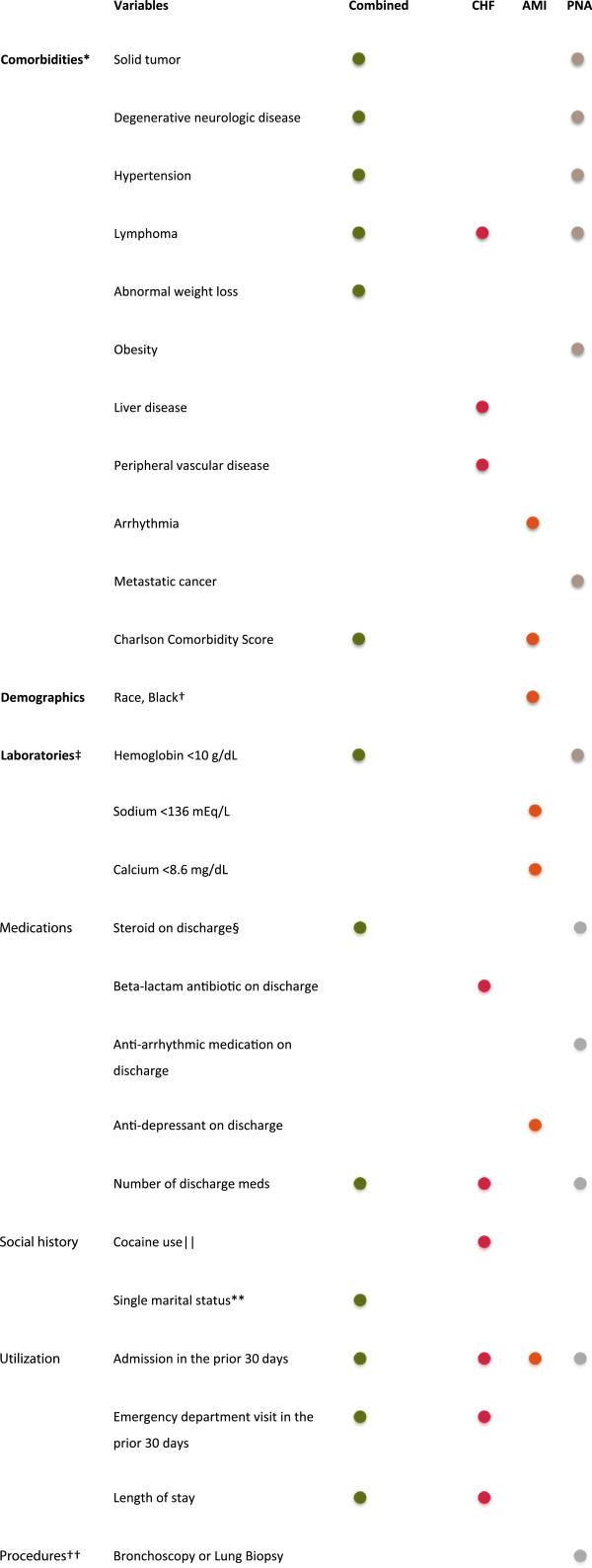
**Variables included in final regression models for each comorbid condition.** *Based on the enhanced ICD-9 coding of the Elixhauser comorbidity classification [29]. Hypertension combines hypertension, uncomplicated with complicated. Only used data from index encounter. ^†^Versus not black. ^‡^At least once during the index hospitalization. ^§^Excluding topical steroids. || Documented in the social history. **Single includes single, widowed, divorced and separated. ^††^Using ICD-9 procedure codes during index hospitalization.

We developed four models using logistic regression with stepwise removal. None of the models showed evidence of a lack-of-fit. The AUC for the derivation models ranged from 0.64 to 0.73 (Additional file 1). All models included the variable *prior admission in the last 30 days* as a risk factor for readmission, while three of the four models included *number of discharge medications* and a diagnosis of *lymphoma* (Figure 2). All other variables were included in only one or two models.

#### Validation cohort

**Figure 3:**
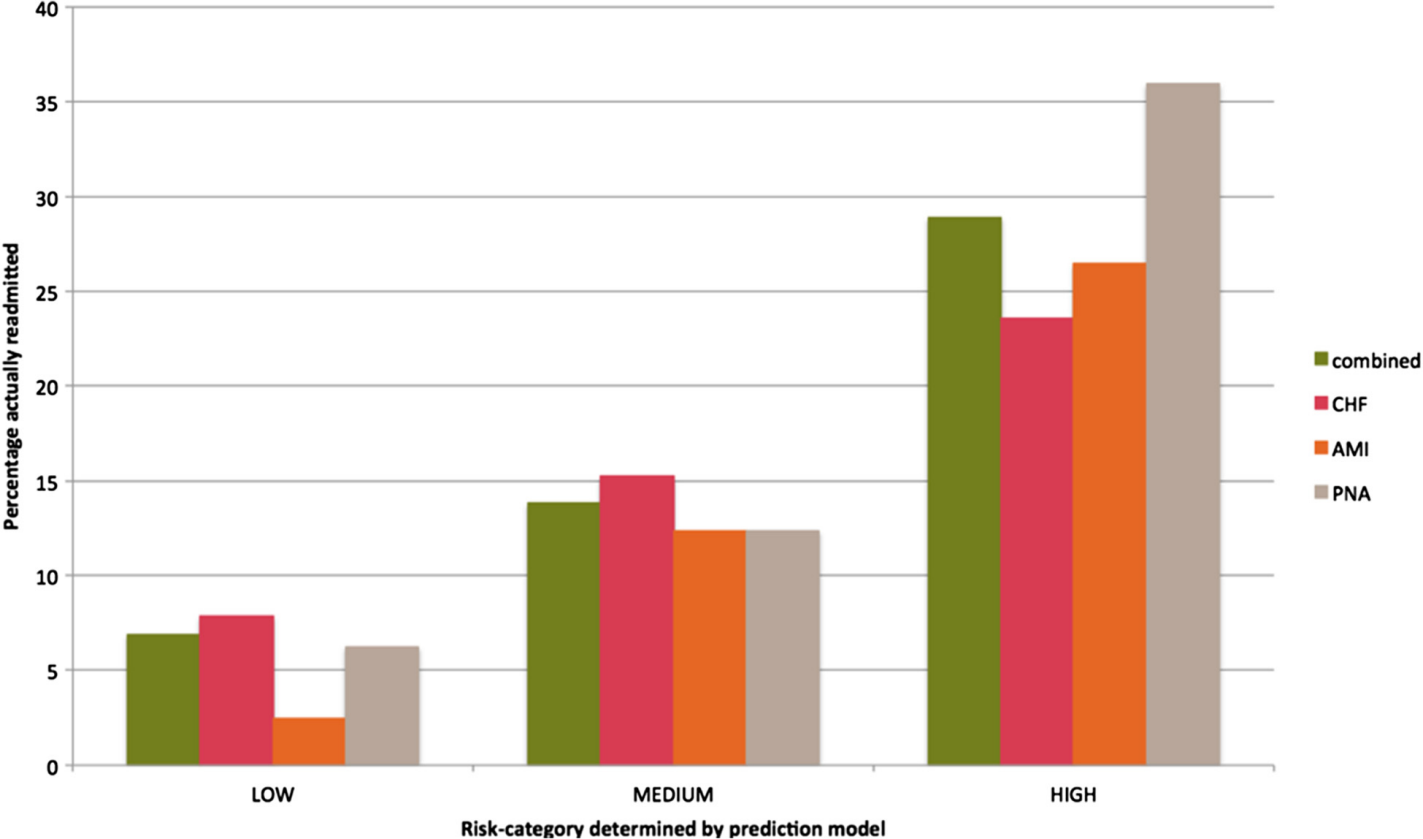
Percentage of patients readmitted in each predicted risk category in the random sample validation cohort.

When the models developed in the derivation cohort were tested on the random sample validation cohort, the AUCs ranged from 0.63 to 0.76 (Additional file 1) and showed no evidence of a lack-of-fit. The model was able to appropriately group patients into high-, medium-, and low-risk groups (Figure 3). When the historical cohort was used for validation, the AUCs were lower, ranging from 0.61 to 0.68. The combined model and AMI model showed no evidence of a lack-of-fit on the historical validation cohort, however, the PNA and CHF models failed to satisfy the goodness-of-fit test. Receiver operating characteristic curves for each of the validation cohorts are available in Additional file 2.

## Discussion

In this study we collected two years of retrospective data in order to determine risk factors for 30-day readmission. We created four prediction models using logistic regression. Our models have moderately good predictive ability in a random sample validation, and less so in a historical cohort. *Previous admission in the last 30 days* was included in all models, while other variables were unique to one or two of the models.

This study adds to the current literature in several ways. First, the models were validated on a contemporary random sample cohort as well as a historical cohort. We found that the models performed much better on the contemporary cohort. This may be due to quality improvement initiatives implemented after the time the historical data were collected. These resulted in lower readmission rates and potentially different risk factors for readmission in the derivation cohort, making the models less generalizable. This highlights the need for models to be updated and trained on current data in order to account for secular trends.

Second, the disease-specific models generally performed better than the combined, suggesting that a disease-specific approach to prediction is superior. This is likely due to the differing characteristics of these conditions, resulting in inconsistent effects of the variables we studied. When these heterogeneous conditions are combined, the resulting model has lower predictive ability for readmission. The exception to this finding was the CHF model, which was the poorest performing model. This may be because CHF is unique in that it is a chronic disease. In the AMI and PNA cohorts there are otherwise healthy patients mixed in with the chronically ill, and our models are able to discriminate between these two groups. The CHF group includes patients who are chronically ill by definition, and so their risk factors for readmission are more difficult to identify using a tool that mainly accounts for comorbidities and hospital and medication utilization. Future plans to improve the CHF model include adding non-clinical risk factors such as neighborhood socioeconomic status and more enriched social history data to the model.

Finally, our models focused on only encounter level variables, not including ICD-9-CM that had been previously recorded or lab values from previous admissions. This was done for two reasons. One was to avoid biasing the model toward patients who receive all their care at OSUWMC and would therefore have more recorded historical data. The other was so that these models could be turned into a tool that resides in the EHR, in order to predict readmissions at the point-of-care. Several barriers need to be overcome in order to integrate the predictive model into the EHR, including mapping our variables to appropriate fields in the new EHR platform, identifying the targeted cohort for the alert, deciding who should get the alert and when the alert would be triggered. In light of our findings in this study, we also acknowledge it is critical for these models to be dynamic, prospectively trained, and able to adjust to changes in patient population, improved discharge procedures, and temporal trends in treatment.

It was not the goal of this study to identify specific risk factors for readmission, but rather to develop a model that predicted readmission. Nevertheless, it is valuable to examine the variables that we found to be markers of readmission in light of previous research.

Hospital utilization was important in all of the models. *Length of stay* and *ED visit* or *inpatient visit in the last 30 days* were common to several of the models. This trend has been seen in many other studies [7,32], likely reflecting the fact that a large percentage of the hospital resources in our country are utilized by a small percentage of patients [10]. In previous studies, demographic factors such as marital status, age and gender have been shown to be predictive of 30-day readmission [21,33,34]. *Single marital status* was a predictor in the combined model, which may suggest a lack of power to detect a significant finding in the smaller cohorts. Gender was not a risk factor in any model nor was age.

Comorbidities have been important predictors of readmission in other studies, specifically diabetes and renal failure [35-37]. The comorbidities that we found to be predictive were diverse and included *other neurologic disease*, cancer, and *abnormal weight loss*. Surprisingly, renal failure, diabetes or other chronic medical issues were not predictive of readmission in this cohort. This may be because earlier initiatives had focused on these high-risk patients, and may have decreased the readmission rate for this group.

Several studies have pointed to the increased risk of readmission due to certain medications. In a recent study by Budnitz et al., adverse drug reactions due to warfarin, insulin, oral hypoglycemic and antiplatelet agents accounted for a significant proportion of hospitalizations [38]. We did not find an association with these medications; however, the number of medications prescribed on discharge was included in three of our models, likely reflecting the risk of polypharmacy [39].

There are several limitations to our study. The EHR platform that was present when the derivation cohort was drawn did not adequately collect social history data such as smoking status (67% missing) and living situation (55% missing). We are working to ensure that our new EHR platform has more complete data on these risk factors. We were also limited to our own medical system’s data. Same-hospital readmission is thought to occur in only 80% of cases [40]. This means we could have misclassified patients if they were readmitted elsewhere. Similarly, if a patient was initially admitted at another hospital, we could have erroneously classified their readmission as an index admission at our institution or misclassified them for the variable *admission in the previous 30 days*. A risk period of 30 days is an arbitrary cutoff and motivated by CMS guidelines. It may be more meaningful to know which patients are going to return to the hospital in the first few days after discharge. We are working to develop cox-proportional hazard models as a next step. Although these data are from one hospital system, and the same predictors may not be risk factors at other large referral centers, the methodology we used to develop the models can be used in other settings.

This was a retrospective study with a goal to create a prediction model. The variables that we found to be significant are likely markers for high-risk patients, but are not necessarily risk factors in themselves and thus should not be highlighted as targets for intervention. A reasonable use for this type of model would be to flag high risk patients within the EHR for prespecified readmission reduction interventions which would not be feasible to roll out to all hospitalized patients, either due to cost or person-time. As a next step, we will attempt to address the aforementioned limitations. Results from this study will inform changes in the EHR system, and improvements in data collection methods are currently underway. Once our models are integrated in the EHR, we will prospectively train the model to continuously refine and improve its predictive accuracy. We plan to enrich these data with information from other sources, including outpatient pharmacy data, clinic visits that occur outside of the OSUWMC, and payer data. We are also exploring further statistical analysis and artificial intelligence approaches to complement our logistic regression methodology.

## Conclusion

Using two years of retrospective administrative and clinical data from our EHR, we developed models to identify patients at risk for 30-day hospital readmission. Our study suggests that disease-specific readmission prediction models are better able to distinguish high-risk patients from low-risk patients. We can use these models in our hospital system to identify inpatients that are at high risk, and target interventions to prevent readmissions. As we continue to train our models prospectively and augment our analysis with additional data sources and methods, we believe we will develop even more accurate prediction models.

## Competing interests

The authors declare that they have no competing interests.

## Authors’ contributions

All authors listed made significant contributions to the conception and design of the study. JW, CS and CH additionally contributed to data acquisition and analysis. All authors were involved substantially in the interpretation of the data. All authors were involved in the drafting and revising of the article, and all gave final approval of the article to be published.

## Pre-publication history

The pre-publication history for this paper can be accessed here:

http://www.biomedcentral.com/1472-6947/14/65/prepub

## Supplementary Material

Additional file 1 Included variables and adjusted odds ratios of readmission in 30 days. Click here for file

Additional file 2 Further details of the performance of readmission models. Click here for file
