# Supplementary material for: Diagnosis-specific readmission risk prediction using electronic health data: a retrospective cohort study
Source: BMC Med Inform Decis Mak. 2014 Aug 4;14:65. doi: 10.1186/1472-6947-14-65 (PMC4136398; doi:10.1186/1472-6947-14-65)
Supplement: Additional file 1 — Included variables and adjusted odds ratios of readmission in 30 days. [file 1472-6947-14-65-S1.doc]

**Additional file 1: Included variables and adjusted odds ratios of readmission in 30 days**

**2a: CHF Model:**

derivation n= 1202, AUC=0.64, GOF: p 0.71

random sample validation n= 300, AUC = 0.63, GOF: p =0.92

historical validation n= 610, AUC= 0.61, GOF: p <0.05

|  | Adjusted model* | |
| --- | --- | --- |
| Variables | **OR** | **95%CI** |
| Inpatient visit in the last 30 days | 1.91 | 1.22-2.99 |
| ED visit in last 30 days | 2.84 | 1.39-5.79 |
| Log length of stay | 1.25 | 1.03-1.51 |
| Cocaine use† | 3.03 | 1.10-8.37 |
| Lymphoma‡ | 2.88 | 1.12-7.39 |
| Peripheral vascular disease‡ | 1.59 | 0.99-2.55 |
| Liver disease‡ | 1.71 | 0.90-3.25 |
| Beta-lactam antibiotic | 1.86 | 0.95-3.65 |
| Number of discharge medications | 1.03 | 1.00-1.06 |

**2b: PNA model**

derivation n= 1042, AUC=0.73, GOF: p 0.16

random sample validation n= 258, AUC = 0.73, GOF: p =0.18

historical validation n= 552, AUC= 0.66, GOF: p <0.05

|  | Adjusted model* | |
| --- | --- | --- |
| Variables | **OR** | **95%CI** |
| Inpatient visit in the last 30 days | 1.91 | 1.27-2.87 |
| Solid tumor without metastasis‡ | 1.99 | 1.13-3.49 |
| Other neurologic disorder‡ | 1.97 | 1.14-3.39 |
| Obesity‡ | 0.51 | 0.25-1.02 |
| Hypertension‡ | 1.38 | 0.96-2.00 |
| Lymphoma‡ | 1.95 | 0.99-3.81 |
| Metastatic cancer‡ | 1.77 | 0.91-3.43 |
| Anti-arrhythmic medication | 2.37 | 1.15-4.86 |
| Steroid§ | 1.47 | 1.01-2.15 |
| Number of discharge medications | 1.03 | 1.00-1.06 |
| Hemoglobin <10g/dL|| | 1.69 | 1.18-2.42 |
| Bronchoscopy or lung biopsy** | 1.50 | 0.96-2.34 |

**2c: AMI model**

derivation n= 936, AUC=0.70, GOF: p 0.39

random sample validation n= 230, AUC = 0.76, GOF: p =0.77

historical validation n= 594, AUC= 0.66, GOF: p =.22

|  | Adjusted model* | |
| --- | --- | --- |
| Variables | **OR** | **95%CI** |
| Inpatient visit in last 30 days | 2.98 | 1.56-5.69 |
| Race, Black†† | 1.72 | 1.08-2.75 |
| Arrhythmia | 1.49 | 0.99-2.23 |
| Charlson comorbidity score | 1.16 | 1.05-1.27 |
| Anti-depressant use | 1.65 | 1.07-2.56 |
| Calcium<8.6 mg/dL|| | 1.77 | 1.16-2.71 |
| Sodium <136 mEq/L|| | 1.49 | 0.98-2.25 |

**2d:Combined model**

derivation n= 3572, AUC=0.68, GOF: p 0.17

random sample validation n= 396, AUC = 0.70, GOF: p =0.89

historical validation n= 1756, AUC= 0.68, GOF: p =0.42

|  | Adjusted model* | |
| --- | --- | --- |
| Variables | **OR** | **90%CI** |
| Inpatient visit in last 30 days | 2.00 | 1.55-2.58 |
| ER visit in the last 30 days | 1.69 | 1.15-2.50 |
| Log length of stay | 1.18 | 1.04-1.35 |
| Marital status, Single | 1.25 | 1.03-1.51 |
| Hypertension‡ | 1.27 | 1.01-1.58 |
| Lymphoma‡ | 1.94 | 1.17-3.21 |
| Solid tumor without metastasis‡ | 2.01 | 1.37-2.93 |
| Other neurological disorder‡ | 1.46 | 1.01-2.10 |
| Abnormal weight loss‡ | 1.49 | 1.07-2.08 |
| Charlson comorbidity score | 1.08 | 1.03-1.33 |
| Steroid§ | 1.40 | 1.09-1.79 |
| Number of discharge medications | 1.02 | 1.00-1.04 |
| Hemoglobin <10|| | 1.33 | 1.08-1.65 |

Abbreviations: AUC, area under the receiver operating characteristic curve; GOF, Hosmer-Lemeshow goodness-of-fit test; OR, odds ratio; 95% CI, 95% confidence interval; ED, emergency department; AMI, acute myocardial infarction; CHF, congestive heart failure; PNA, pneumonia.

*Adjusted for all other covariates in the model

†Documented in the social history

‡Based on the enhanced ICD-9 coding of the Elixhauser comorbidity classification . Hypertension combines hypertension, uncomplicated with complicated. Only used data from index encounter.

§Excluding topical steroids

||At least once during the index hospitalization

**Using ICD-9 procedure codes during index hospitalization

††Versus not black
