# Supplementary material for: Diagnosis-specific readmission risk prediction using electronic health data: a retrospective cohort study
Source: BMC Med Inform Decis Mak. 2014 Aug 4;14:65. doi: 10.1186/1472-6947-14-65 (PMC4136398; doi:10.1186/1472-6947-14-65)

Additional file 2: Figure 1

Combined model

Random sample validation:

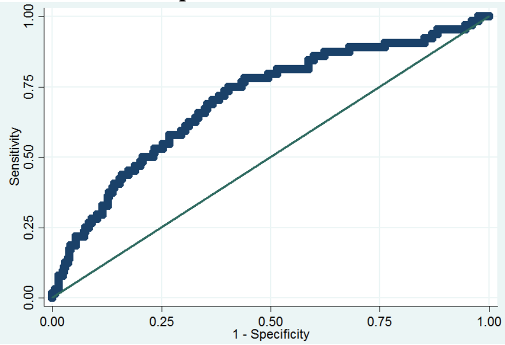

Historical validation:

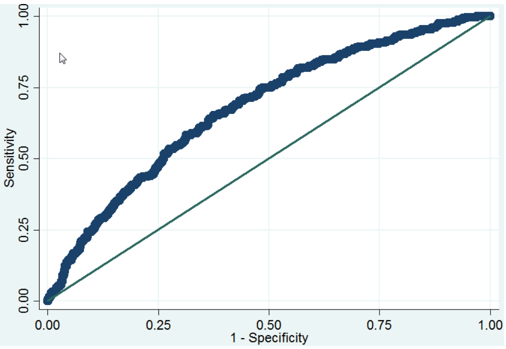

Pneumonia model

Random sample validation:

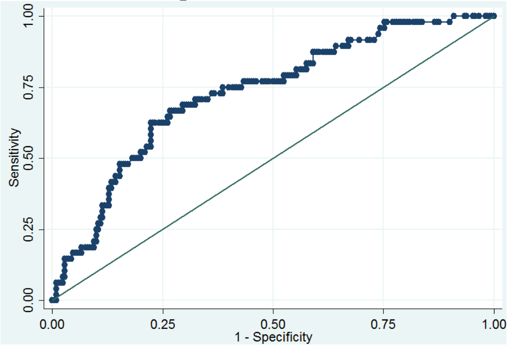

Historical validation

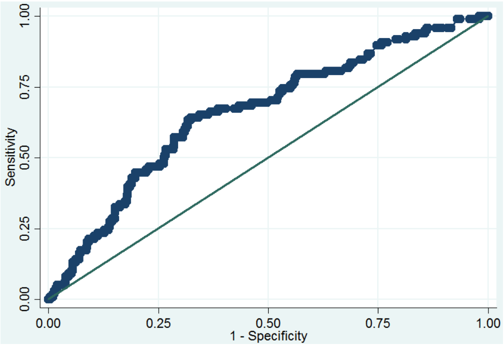

Congestive heart failure model

Random sample validation:

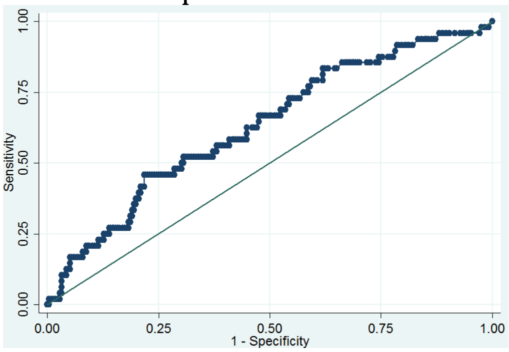

Historical validation:

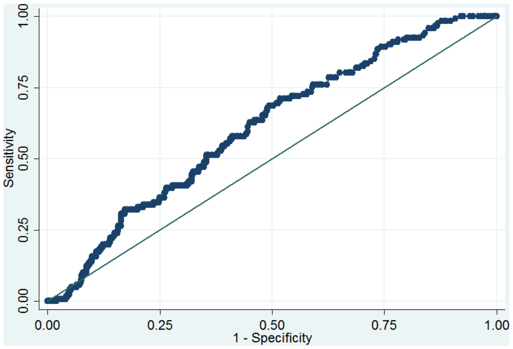

Acute myocardial infarction model

Random sample validation:

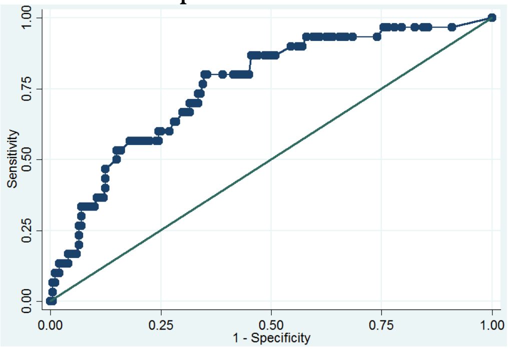

Historical validation:

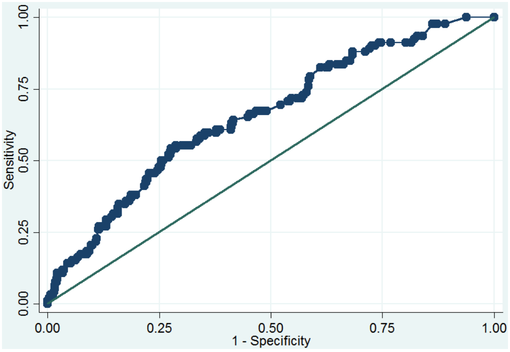

Additional file 2: Figure 2: Decile of risk versus actual readmission rate for random sample validation (A) and historical validation (B)

A:

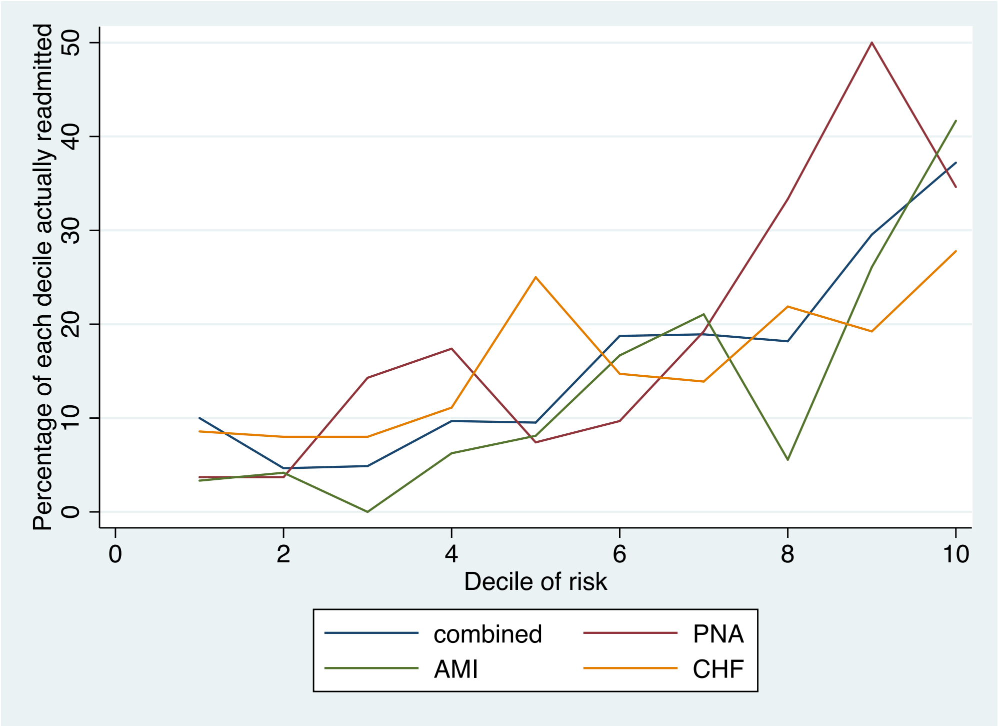

B:

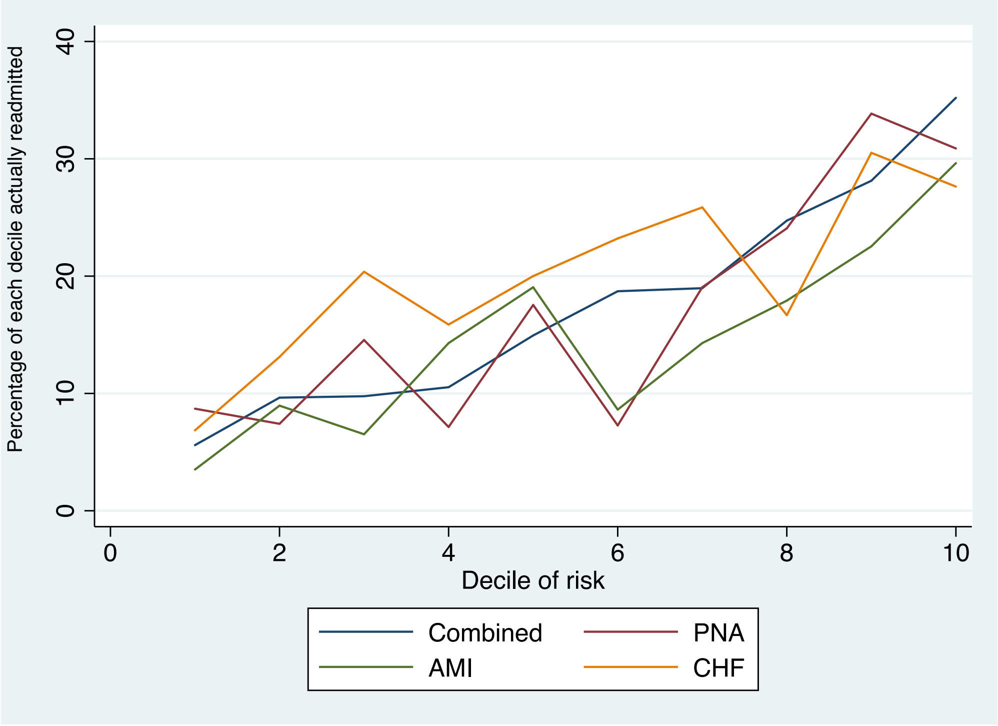

Supplement: Additional file 2 — Further details of the performance of readmission models. [file 1472-6947-14-65-S2.pdf]
